# Fabrication of UV-Curable Polysiloxane Coating with Tunable Refractive Index Based on Controllable Hydrolysis

**DOI:** 10.3390/nano13131985

**Published:** 2023-06-30

**Authors:** Hong-Lan Huang, Qi-Kai Shi, Yan Deng, Xiang-Yang Lei, Qing-Huang Zhang, Jin-Ju Chen, Xue-Ran Deng

**Affiliations:** 1 Research Center of Laser Fusion, China Academy of Engineering Physics, Mianyang 621900, China; mrhuanghonglan@163.com (H.-L.H.); aokole@126.com (Q.-K.S.); dyswallow2001@sohu.com (Y.D.); leixiangyang2@163.com (X.-Y.L.); zhangqh506@163.com (Q.-H.Z.); 2 School of Materials and Energy, University of Electronic Science and Technology of China, Chengdu 610054, China

**Keywords:** controllable hydrolysis, UV-curable polysiloxane coating, refractive index modulation, laser damage resistance, mechanical property

## Abstract

In order to improve laser transmission efficiency at 1053 nm and 527 nm, a potassium deuterium phosphate (DKDP) crystal (a key component of high-power laser systems) needs a bi-layer antireflection coating system on its incident surface. UV-curable polysiloxane coatings with a refractive index varying from 1.500 to 1.485 were prepared through the polycondensation of a methacryloxy propyl trimethoxylsilane (MPS) monomer with a controllable degree of hydrolysis. Additionally, the influence rule of the coating structure on the refractive index was intensively studied, and the primary factors that dominate the hydrolysis process were discussed. Further refractive index adjustment was achieved using only a small amount of dopant based on the polysiloxane coating with refractive index of 1.485, allowing for high antireflection of the bi-layer coating system at desired wavelengths to be achieved. In addition, high laser damage resistance and remarkable mechanical properties of the coating were simultaneously realized through the incorporation of a minor quantity of dopants, which benefited from the successful modulation of the intrinsic refractive index of the polysiloxane coating.

## 1. Introduction

Controllable fusion provides a new potential method for realizing clean energy. The process can be driven by a high-power laser system [1,2], in which the laser frequency tripler (composed of potassium deuterium phosphate (DKDP) crystal and an antireflection coating) is a key optical component. In order to improve the laser transmission efficiency at both 1053 nm and 527 nm and maintain high laser damage resistance, the antireflection coating conventionally consists of a polysiloxane layer (with a high refractive index) and a porous SiO_2_ layer (with a low refractive index) [3,4,5]. The sol–gel technique is a favorable method for preparing such coatings due to its advantages, like its low cost, high chemical purity, high preparation efficiency, and strong resistance to laser damage [6,7,8], compared with other methods [9,10,11]. However, sol–gel-prepared polysiloxane coatings need to be cured at 140 °C (which is higher than the phase transition temperature of DKDP crystal [12]), and its intrinsic refractive index is higher (around 1.5 [13,14]) than the designed value (around 1.37) to achieve high transmission at both 1053 nm and 527 nm.

The literature has revealed that UV curing is a helpful method for reducing curing temperatures [15,16,17,18]. In addition, methacryloxy propyl trimethoxylsilane (MPS) is an appropriate raw material for the preparation of polysiloxane coating due to its UV-curable group. As for the control of a coating’s refractive index, scholars have found that changing the crosslinking structure of a coating can lead to the variation of the refractive index [19]. However, MPS with varying degrees of hydrolysis must be prepared first, in order to obtain polysiloxane coatings with different crosslinking structures. It has been reported that siloxane can be hydrolyzed using acid [20] or alkali [21] as catalysis, but it tends to spontaneously polycondense in an alkaline environment [21]. Moreover, organic acids [22,23], tetrahydrofuran (THF) [24], and the use of a lower hydrolysis temperature [25] have been proven to effectively inhibit the spontaneous polycondensation of silanols. Therefore, it is prudent to carry out the synthesis process in an ice bath environment using an organic acid as a catalyst and THF as a polymerization inhibitor. At present, there are no clear conclusions regarding the growth-driving factors of the crosslinking structure in polysiloxane coatings. In addition, the influence of the crosslinking structure on the refractive index of polysiloxane coatings also remains unclear. So, more research is needed to clarify the growth law and derived properties of polysiloxane coatings with different crosslinking structures.

In this work, polysiloxane coatings with different crosslinking structures were prepared using MPS monomers with varying hydrolysis degrees, and the influence of the crosslinking structure on the refractive index was discussed. The spontaneous polycondensation of Si-OH was found to be effectively inhibited by adding THF and reducing the hydrolysis temperature. In addition, a bi-layer antireflection coating system with high transmission at both 1053 nm and 527 nm was successfully fabricated using the polysiloxane coating after the precise modulation of the refractive index and thickness.

## 2. Materials and Methods

### 2.1. Materials

MPS was obtained from Aladdin Chemistry. THF and sec-butanol were purchased from Alfa-Aesar. Acetic acid (CH_3_COOH) and n-heptane were supplied by Chengdu Chron Chemicals Co., Ltd., Chengdu, China. 2-hydroxy-2-methylpropiophenone was purchased from Shanghai Mclean Biochemical Technology Co., Ltd., Shanghai, China. Deionized water was produced using a water purifying equipment. All chemicals were used without further purification.

### 2.2. Hydrolysis of MPS

The concentrations of the reactants in the hydrolysis system under different experimental conditions are shown in Table 1. All reactants were placed in a flask to be hydrolyzed with the assistance of a magnetic stirrer. It should be noted that MPS needed to be mixed with THF evenly before the CH_3_COOH was added.

The hydrolysis reactions described above were carried out at room temperature. In addition, the hydrolysis of MPS in a water-rich system was also carried out in an ice bath environment with the same parameters. The experimental group hydrolyzed at room temperature was labeled RT, and the experimental group hydrolyzed in the ice bath environment was labeled IT.

### 2.3. Preparation of Polysiloxane Prepolymer

Direct polycondensation: A total of 50 mL of hydrolysate with different degrees of hydrolysis was stirred (800 rpm) and heated (~80 °C) for 1 h, 2 h, and 3 h to prepare prepolymer.

Polycondensation after removal of water: A total of 50 mL hydrolysate and a 20 g molecular sieve were placed in a sealed flask. Then, the mixture was kept at 5 °C for 24 h in order to remove excess water in hydrolysate. After the removal of water, the products were heated to achieve polycondensation under the same parameters as mentioned above.

Polycondensation after the removal of THF: After the water removal step, 50 mL of the product was placed in a fume hood for 2 h to remove THF via air flow. Then, the product was heated to achieve polycondensation under the same parameters as mentioned above.

### 2.4. Preparation of Porous SiO_2_ Nanoparticles

The preparation of porous SiO_2_ nanoparticles has been described in our previous work [14]. There are two main usage of porous SiO_2_ nanoparticle regarding the application: (1) The refractive index of a polysiloxane coating can be accurately modulated by embedding porous SiO_2_ nanoparticles. (2) The coating with a lower refractive index in the bi-layer antireflection coating system is composed of porous SiO_2_ nanoparticles.

### 2.5. Preparation of Polysiloxane Coating

Prepolymers with different crosslinking structures (1 g), sec-butanol (20 g), n-heptane (10 g), and 2-hydroxy-2-methyl-1-phenylacetone (1 g) were placed in a flask, and this mixture was blended for 12 h at a stirring rate of 600 rpm at room temperature to prepare sols for coating. 1 mL of the resulting sol was dropped onto a silicon wafer, and the coating was fabricated after spin coating at 550 rpm for 30 s. Then, the obtained coating was dried for 2 h at room temperature to remove the solvent completely. Finally, the polysiloxane coating was irradiated under an ultraviolet lamp at 100 mW/cm^2^ for 20 s to be completely cured.

In order to precisely modulate the refractive index of the polysiloxane coating, different quantities of porous SiO_2_ nanoparticles were added into the previous sols to endow extra porosity. The mass ratio of the prepolymer, sec-butanol, n-heptane, and 2-hydroxy-2-methyl-1-phenylacetone was fixed at 1:60:30:1, and the mass ratio of porous SiO_2_ was varied from 50 to 100. Similar to the spin-coating process applied for the polysiloxane coating, 1 mL of composite sol with a different mass ratio of SiO_2_ nanopaticle was dropped onto a silicon wafer. Then, a homogeneous SiO_2_-embedded polysiloxane coating was obtained at a speed of 800 rpm applied for 30 s. Finally, this coating was cured using an ultraviolet curing lamp, as mentioned above.

### 2.6. Preparation of bi-Layer Antireflection Coating System

**Figure 1:**
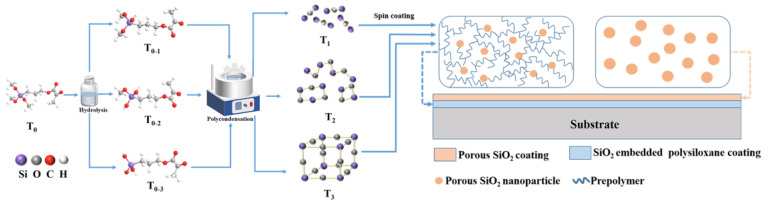
Preparation process of the bi-layer antireflection coating system.

A SiO_2_-embedded polysiloxane coating was prepared and cured over a DKDP substrate (5 cm × 5 cm × 1 cm); then, a layer of porous SiO_2_ coating was spin-coated over the cured polysiloxane coating at a speed of 660 rpm for 30 s. Further curing at 100 °C for 6 h was applied to form the bi-layer antireflection coating system, as shown in Figure 1.

### 2.7. Characterizations

Compositional and structural information on the hydrolysates and prepolymers was obtained via nuclear magnetic resonance spectroscopy (NMR). The functional groups of the hydrolysates and pretreated products were detected using Fourier transform infrared spectroscopy (FTIR). A viscometer, a moisture tester, and gel permeation chromatography (GPC) were used to characterize the viscosity, moisture content, and average molecular weight of the prepolymer, respectively. The refractive index and transmittance of the coating were measured using an ellipsometer and an ultraviolet-visible spectrophotometer, respectively. The hardness and elastic modulus of the different coatings were characterized via nanoindentation. In addition, the laser-induced damage threshold (LIDT) of the coatings was measured using a self-made laser damage platform (whose beam diameter was about 610 µm, repetition frequency was 1 Hz, and measurement error was within 7% at 1064 nm) using S-on-1 mode (the laser beam was focused at a single spot on the coating, and the laser energy was gradually increased until the coating was damaged). At least 100 sampling spots were tested for each sample.

## 3. Results

### 3.1. Study on the Controllable Hydrolysis of MPS Monomer

**Figure 2:**
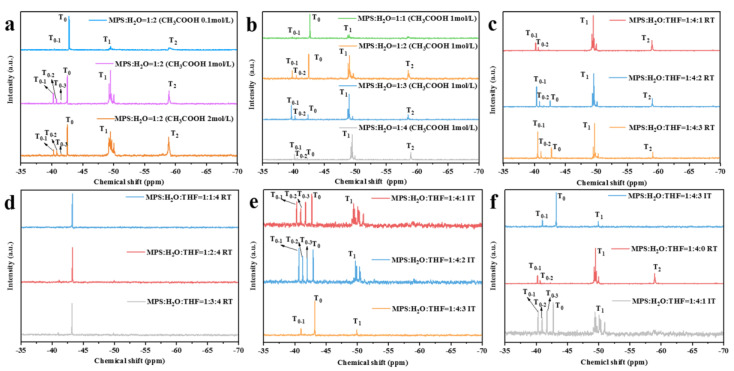
^29^Si NMR spectra of hydrolysis products of MPS: (**a**) different CH_3_COOH concentrations; (**b**) different water ratios; (**c**) excessive amount of water at room temperature; (**d**) excessive amount of THF at room temperature; (**e**) excessive amount of water at ice bath temperature; (**f**) MPS with different degrees of hydrolysis.

In order to determine the proper conditions for realizing the controllable hydrolysis of MPS monomers, the effects of crucial factors were individually studied. The information of the hydrolysates is analyzed via ^29^Si NMR spectra and shown in Figure 2 [26,27]. The unhydrolyzed MPS monomer is represented by T_0_, and the monomers holding one, two, and three hydroxyl groups after hydrolysis are represented by T_0-1_, T_0-2_, and T_0-3_, respectively. The polymers with one, two, and three silicon atoms linked to the central silicon atom are labeled T_1_, T_2_, and T_3_, respectively.

In Figure 2a, the peak near −40 ppm corresponds to T_0_, and the peaks near −50 ppm, −60 ppm, and −70 ppm correspond to T_1_, T_2_, and T_3_, respectively. In addition, the resonance peaks of T_0-3_, T_0-2_, and T_0-1_ appear in sequence near the T_0_ peak [26]. It was found that when the concentration of CH_3_COOH is 1 mol/L, the intensity of the T_0-1_, T_0-2_, and T_0-3_ peaks are relatively strong compared with that of T_0_. While the concentration of CH_3_COOOH was 0.1 mol/L, the concentration of H^+^ was low in the hydrolysis system. The alkoxy group cannot be effectively attacked by protons, so a large amount of MPS cannot be hydrolyzed. Moreover, when the concentration of CH_3_COOH increases to 2 mol/L, a large amount of CH_3_COO^−^ hinders the nucleophilic attack of water molecules, which prolongs the hydrolysis reaction and makes some silanols spontaneously condense, causing the intensity of the T_0-1_, T_0-2_, and T_0-3_ peaks to decrease significantly. These results prove that a concentration of CH_3_COOH of about 1 mol/L is optimal for obtaining an MPS hydrolysate with different degrees of hydrolysis.

It can be seen from Figure 2b that when the molar ratio of MPS and H_2_O is 1:1, the intensity of T_0-1_ in the hydrolysis product is weak, while the intensity of T_0_ is strong. The results show that only a few MPS monomers hydrolyzed. After increasing the molar ratio of H_2_O, the intensity of T_0_ gradually decreased, and the intensity of T_1_ and T_2_ gradually increased. Accordingly, it can be concluded that insufficient water could impede the hydrolysis of MPS monomers, while an inhibitor is necessary to limit the spontaneous polycondensation of hydrolyzed monomers.

THF was used as an inhibitor in the water-rich (MPS:H_2_O = 1:4) system, and the corresponding NMR results are given in Figure 2c. When the molar ratio of THF was 1, there were no unhydrolyzed monomers (T_0_), but most of the silanols existed in the form of T_1_. When the molar ratio of THF continuously increased, the T_0_ peak appeared, and the intensity of T_0-1_ and T_0-2_ gradually increased. This result reveals that the inhibitor can limit spontaneous polycondensation and aids the acquisition of MPS monomers with different degrees of hydrolysis. However, as indicated in the NMR result regarding hydrolysis under an excessive concentration of THF (MPS:THF = 1:4) shown in Figure 2d, it was found that only the T_0_ peak can be observed regardless of the water ratio, which indicates that MPS cannot be hydrolyzed in the THF-rich system.

Figure 2e presents the ^29^Si NMR spectrum of the MPS hydrolysis products under ice bath conditions using THF as an inhibitor. It shows that when the molar ratio of THF is from 1 to 2, T_0-1_, T_0-2_, and T_0-3_ can be all obtained with considerable intensity, while the intensity of T_1_ is restrained effectively. However, when the molar ratio of THF reaches 3, the T_0-2_ and T_0-3_ peaks disappear. The hydrolysis products contain only T_0_, T_0-1_, and a small amount of T_1_. This result means that both the hydrolysis and spontaneous polycondensation of MPS are limited under the combined effect of an ice bath and an inhibitor.

Since hydrolysis and spontaneous polycondensation constitute a complicated and restricting process, an MPS monomer with a specific hydrolyzed structure (such as pure T_0-x_, x = 1, 2, or 3) is difficult to fabricate. Therefore, three types of MPS monomers with different degrees of hydrolysis were chosen for analysis, and the corresponding NMR spectra are depicted in Figure 2f. When the ratio of MPS:H_2_O:THF was 1:4:0 (RT), T_0-1_, T_0-2_ but T_0-3_ was observed in the hydrolysate. When the ratio of MPS:H_2_O:THF was 1:4:1 (IT), the concentrations of T_0-1_ and T_0-3_ were relatively high. Moreover, the content of T_0-2_ was lower than that of T_0-1_ and T_0-3_ in the hydrolysate. When the ratio of MPS:H_2_O:THF was 1:4:3 (IT), only T_0-1_ existed in the hydrolysate. These three types of hydrolysates are considered relatively typical and were used in further studies on the preparation of MPS prepolymers and polysiloxane coatings.

**Figure 3:**
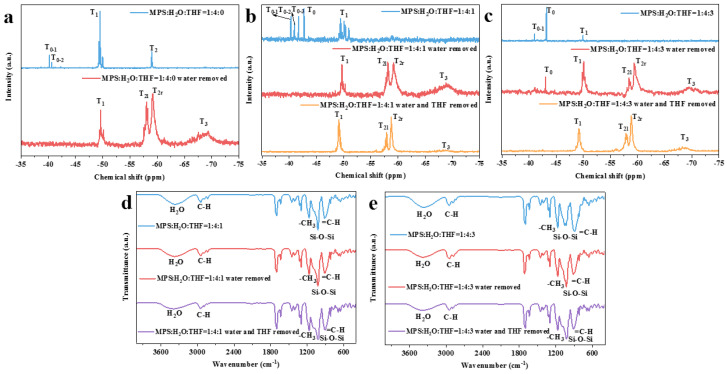
Structural information on pretreated products: (**a**) ^29^Si NMR spectra of MPS:H_2_O:THF = 1:4:0 (RT); (**b**) ^29^Si NMR spectra of MPS:H_2_O:THF = 1:4:1 (IT); (**c**) ^29^Si NMR spectra of MPS:H_2_O:THF = 1:4:3 (IT); (**d**) FTIR spectra of MPS:H_2_O:THF = 1:4:1 (IT); (**e**) FTIR spectra of MPS:H_2_O:THF = 1:4:3 (IT).

The compositions of the hydrolysates after pretreatment (namely, the removal of water and THF) were characterized according to their ^29^Si NMR spectra. After the removal of water (Figure 3a), the T_0_ peak disappeared, and the T_2_ peak split into two sub-peaks, namely, T_2l_ and T_2r_, which correspond to network and long-chain structures, respectively. Meanwhile, a three-dimensional polymer (T_3_) appeared. These results imply that removing water can promote the polycondensation reaction and thus the formation of more complex structures. With the further removal of THF (Figure 3b,c), the intensity of T_2r_ in the hydrolysate increases prominently, indicating that silanols are more prone to forming chain structures via spontaneous polycondensation in the THF-free system.

After the removal of water, the FTIR spectra (Figure 3d,e) revealed that the intensity and width of the Si-O-Si vibration peak in the 1100 cm^−1^ to 980 cm^−1^ range of different THF ratios both increase, which proves that removing water can promote the spontaneous polycondensation of the hydrolyzed monomer. This conclusion is consistent with the ^29^Si NMR results. Furthermore, the width of the Si-O-Si bond absorption peak in the FTIR spectra of the hydrolysate increased further after removing THF, which indicates that the removal of THF can also promote the spontaneous condensation of a hydrolysate.

### 3.2. Preparation of Prepolymers with Different Crosslinking Structures

**Figure 4:**
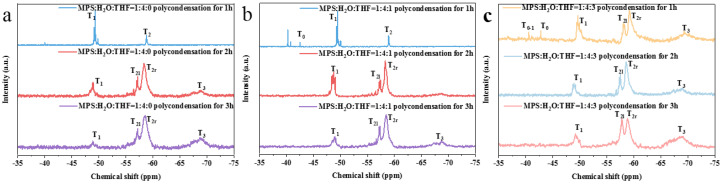
^29^Si NMR spectra of prepolymer prepared via the direct polycondensation of hydrolysate: (**a**) MPS:H_2_O:THF = 1:4:0 (RT); (**b**) MPS:H_2_O:THF = 1:4:1 (IT); (**c**) MPS:H_2_O:THF = 1:4:3 (IT).

The preparation of the prepolymer was further carried out using the previously chosen groups of controllable hydrolyzed monomers (e.g. MPS:H_2_O:THF = 1:4:0 (RT), MPS:H_2_O:THF = 1:4:1 (IT), and MPS:H_2_O:THF = 1:4:3 (IT)). When the hydrolysate of MPS:H_2_O:THF = 1:4:0 (RT) directly polycondensed (Figure 4a) for 1 h, the prepolymer was mainly composed of short-chain polymers (T_1_) and a small quantity of long-chain polymers (T_2r_). After 2 h of polycondensation, the number of short-chain structures decreased, while network (T_2l_) and three-dimensional (T_3_) crosslinking structures appeared. When the polycondensation time was increased to 3 h, the number of short-chain structures in the prepolymer continued to decrease. Meanwhile, the number of three-dimensional polymers increased. These results show that the extension of the polycondensation time will increase the degree of polymerization. The NMR results of the other two groups of prepolymers (Figure 4b,c) also confirm this conclusion.

Compared with the NMR results regarding the prepolymer after 3 h of polycondensation, the three-dimensional structural content of the prepolymer (MPS:H_2_O:THF = 1:4:1 (IT)) decreased, indicating that a small amount of THF inhibits the growth of three-dimensional crosslinking polymers. The prepolymer with a reactant ratio of MPS:H_2_O:THF = 1:4:3 (IT) had higher quantities of network and three-dimensional structures than those of the other two kinds of prepolymer. This is because the mixture of THF and water has a lower boiling point than that of pure water, which makes THF evaporate rapidly in the early stage of polycondensation and allows silanol to more easily form a three-dimensional crosslinking structure without the protection of THF.

**Figure 5:**
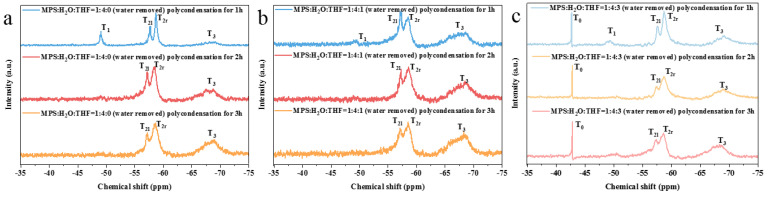
^29^Si NMR spectra of prepolymer prepared through the polycondensation of hydrolysate after the removal of water: (**a**) MPS:H_2_O:THF = 1:4:0 (RT); (**b**) MPS:H_2_O:THF = 1:4:1 (IT); (**c**) MPS:H_2_O:THF = 1:4:3 (IT).

After the removal of water, the hydrolysate was heated for polycondensation. For the prepolymer with the reactant ratio of MPS:H_2_O:THF = 1:4:0 (RT) (Figure 5a), considerable long-chain polymers, network polymers, and short-chain polymers were observed after 1 h of polycondensation. After 2 h of polycondensation, the short-chain polymers disappeared; meanwhile, the number of network polymers increased, and a small quantity of three-dimensional polymers were produced. When the polycondensation time was increased to 3 h, the number of three-dimensional polymers continued to increase.

With the increase in the polycondensation time in the MPS:H_2_O:THF = 1:4:1 (IT) experimental group (Figure 5b), the number of long-chain structures gradually increased. It was assumed that in the early stage of polycondensation, MPS with a high degree of hydrolysis more easily forms a network structure due to the protective effect of THF on Si-OH. However, when THF is volatilized completely, oligomers construct long-chain structures more easily.

After being polycondensed for 3 h, the prepolymer with the reactant ratio of MPS:H_2_O:THF = 1:4:1 (IT) had a higher three-dimensional polymer content than that of MPS:H_2_O:THF = 1:4:0 (RT). This finding may be ascribed to the fact that T_0-2_ and T_0-3_ are more prone to forming network and three-dimensional structures. In addition, due to the low degree of hydrolysis (MPS:H_2_O:THF = 1:4:3 (IT)), the prepolymer contains a large number of unhydrolyzed MPS monomers. This result is highly consistent with the NMR results regarding the hydrolysates in the pretreatment stage (Figure 3c).

**Figure 6:**
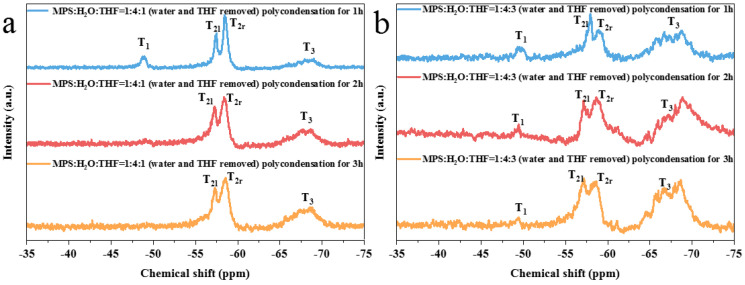
^29^Si NMR spectra of prepolymer prepared via the polycondensation of hydrolysate without excess water and THF: (**a**) MPS:H_2_O:THF = 1:4:1 (IT); (**b**) MPS:H_2_O:THF = 1:4:3 (IT).

Another comparison group of hydrolysates was heated for polycondensation after the removal of water and THF. The crosslinking degree of the prepolymer increased with the increase in the condensation time. The prepolymers of MPS:H_2_O:THF = 1:4:1 (IT) (Figure 6a) and MPS:H_2_O:THF = 1:4:3 (IT) (Figure 6b) still conform to the above rule, and the resulting prepolymers presented the similar structure. It is worth noting that with the same polycondensation time, the prepolymer with the reactant ratio of MPS:H_2_O:THF = 1:4:3 (IT) contains more network and three-dimensional polymers than those of MPS:H_2_O:THF = 1:4:1 (IT). The results show that although the hydrolysate of MPS:H_2_O:THF = 1:4:3 presented the lowest degree of hydrolysis, it more easily forms a high number of crosslinking prepolymers after losing the protective effect of THF.

In summary, pretreatment will promote the formation of prepolymers with higher crosslinking degrees. The prepolymer with the reactant ratio of MPS:H_2_O:THF = 1:4:3 (IT) had the highest number of three-dimensional structures. Furthermore, the prepolymers prepared using the hydrolysates from MPS:H_2_O:THF = 1:4:0 (RT) and MPS:H_2_O:THF = 1:4:1 (IT) had more long-chain structures and network structures, respectively. 

### 3.3. Effect of Different Crosslinking Structures on the Refractive Index of Polysiloxane Coating

**Figure 7:**
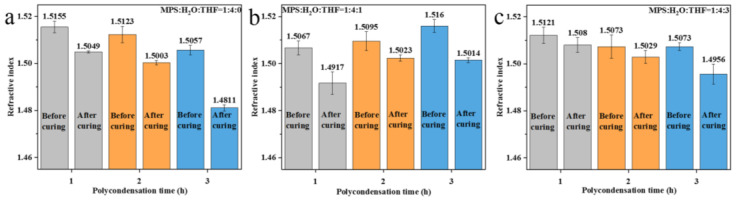
Refractive indices of polysiloxane coatings prepared via direct polycondensation of hydrolysate: (**a**) MPS:H_2_O:THF = 1:4:0 (RT); (**b**) MPS:H_2_O:THF = 1:4:1 (IT); (**c**) MPS:H_2_O:THF = 1:4:3 (IT).

The refractive indices of the polysiloxane coatings prepared using the directly polycondensed prepolymers are shown in Figure 7. Notably, the refractive index of the uncured coating is obviously higher than that of the cured coating, which is due to the influence of residual solvents like sec-butanol and n-heptane (used to adjust the viscosity of the sol for facile coating). So, all the studies presented henceforth were based on the cured coating.

When the polycondensation time of the prepolymer was increased from 1 h to 2 h and 3 h, the refractive index of the polysiloxane coatings (MPS:H_2_O:THF = 1:4:0 (RT)) decreased from 1.5049 to 1.5003 and 1.4811, respectively (Figure 7a). Combined with the NMR results for the prepolymers (Figure 4a), the extension of the polycondensation time will promote further polycondensation from one-dimensional and two-dimensional structures to three-dimensional structures. This result shows that the three-dimensional structures in polysiloxane coatings are helpful to lower the intrinsic refractive index of coatings. As for the coatings prepared with a reactant ratio of MPS:H_2_O:THF = 1:4:1 (IT) (Figure 7b), the coating prepared using the prepolymer with a polycondensation time of 1 h had the lowest refractive index (1.4917). When the polycondensation time was extended to 2 h, the refractive index of the coating increased to 1.5023. During this period, the numbers of MPS monomers and silanol monomers in the prepolymer decrease, while the numbers of chain and network polymers increase (Figure 4b). This result proves that chain and network structures will induce a higher refractive index of polysiloxane coatings. When the polycondensation time was increased to 3 h, the number of three-dimensional structures continued to increase. Meanwhile, the refractive index of the coating further decreased to 1.5014, which confirms the above conclusion that the three-dimensional structure was more helpful in lowering the refractive index of the polysiloxane coating compared with the one-dimensional chain and the two-dimensional network structures. The same variation trend can also be observed in the MPS:H_2_O:THF = 1:4:3 (IT) group (Figure 7c).

**Figure 8:**
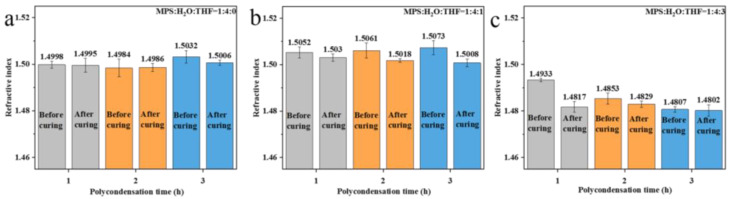
Refractive index of polysiloxane coatings prepared via direct polycondensation of hydrolysate after the removal of water: (**a**) MPS:H_2_O:THF = 1:4:0 (RT); (**b**) MPS:H_2_O:THF = 1:4:1 (IT); (**c**) MPS:H_2_O:THF = 1:4:3 (IT).

The refractive index of the polysiloxane coating prepared using prepolymers after the removal of water was tested, and the results are given in Figure 8. It was found that the refractive index of the coatings prepared with the ratio of MPS:H_2_O:THF = 1:4:0 (RT) was relatively stable (about 1.500). The NMR results show that the crosslinking structure of the prepolymer after the removal of water changes slightly (Figure 5a), which is consistent with the test results of the refractive index of the polysiloxane coatings. The refractive index of the polysiloxane coatings with the reactant ratio of MPS:H_2_O:THF = 1:4:1 (IT) is similar to that of MPS:H_2_O:THF = 1:4:0 (RT). However, the prepolymer prepared using the MPS:H_2_O:THF = 1:4:1 (IT) experimental group has more network structures than MPS:H_2_O:THF = 1:4:0 (RT). When the polycondensation time is same, the refractive index of the coating prepared using the prepolymer with the reactant ratio of MPS:H_2_O:THF = 1:4:1 (IT) is higher than that of MPS:H_2_O:THF = 1:4:0 (RT). As for MPS:H_2_O:THF = 1:4:3 (IT), the refractive index of the polysiloxane coatings is significantly lower (about 1.482) than that of the other two groups. This can also be ascribed to the greater number of three-dimensional crosslinking structures of the coating.

**Figure 9:**
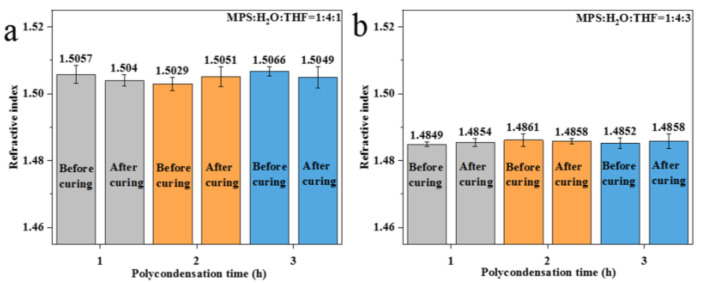
Refractive index of polysiloxane coating prepared via direct polycondensation of hydrolysate with further removal of THF: (**a**) MPS:H_2_O:THF = 1:4:1 (IT); (**b**) MPS:H_2_O:THF = 1:4:3 (IT).

After the further removal of THF, it was found that the refractive indices of the polysiloxane coatings prepared using different polycondensation times remain approximately stable. The intrinsic refractive index of the polysiloxane coatings with different reactant ratios fluctuates around 1.505 (Figure 9a) and 1.485 (Figure 9b), respectively. The increase in the polycondensation time of the prepolymer has a slight effect on the refractive index of the polysiloxane coating, which indicates that the decisive factor affecting the refractive index is the characteristic crosslinking structure.

The test results regarding the prepolymer structure and polysiloxane coating properties obtained through different polycondensation methods are shown in Table 2. The order of the intrinsic refractive indices of the different crosslinking structures, ranked from high to low, is as follows: network structure, chain structure, and three-dimensional structure.

### 3.4. Fabrication of Polysiloxane Coating with Precisely Controlled Refractive Index

**Figure 10:**
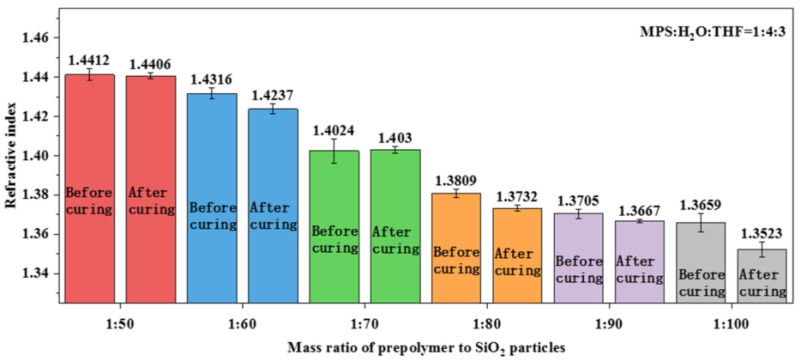
The relationship between SiO_2_ nanoparticle ratio and the refractive index of the coatings.

As the polysiloxane coating prepared using the prepolymer solution with a ratio of MPS:H_2_O:THF = 1:4:3 (IT) has the lowest intrinsic refractive index, it was chosen as the sample for the further study of the bi-layer antireflection coating system. SiO_2_ nanoparticles were added into the prepolymer solution to reduce the refractive index of the cured polysiloxane coating due to its considerable porosity. With the increase in the SiO_2_ nanoparticle content, the refractive index of the polysiloxane coating gradually decreased (Figure 10). When the ratios of the prepolymer to the SiO_2_ nanoparticles were 1:80, 1:90, and 1:100, the refractive index of the polysiloxane coating respectively decreased to 1.3732, 1.3667, and 1.3523, which are suitable values for fabricating the bi-layer antireflection coating system mentioned above.

**Figure 11:**
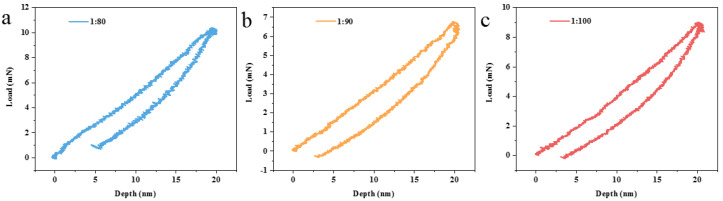
The P–h curves of polysiloxane coatings with different prepolymer to SiO_2_-nanoparticle ratios: (**a**) 1:80; (**b**) 1:90; (**c**) 1:100.

The elastic modulus and hardness of the SiO_2_-embedded polysiloxane coatings with different ratios of prepolymer to SiO_2_ nanoparticles were tested via nanoindentation. The relationship between load (P) and indentation depth (h) is shown in Figure 11. The elastic modulus and hardness of the different coatings were obtained by fitting the load diagram and are listed in Table 3. Among these three groups, coating with the prepolymer to SiO_2_-nanoparticle ratio of 1:80 presented the best mechanical performance. Meanwhile, the elastic modulus and hardness of the coating both decreased with the increase in the number of SiO_2_ nanoparticles. On the other hand, the 1064 nm LIDT of the polysiloxane coating also gradually decreases with the increase in the number of SiO_2_ nanoparticles (Table 4), which is due to the deterioration of the intrinsic structure of the polysiloxane coating and the introduction of more scattering centers.

In summary, the content of SiO_2_ nanoparticles in the polysiloxane coatings is inversely proportional to the refractive index. The refractive index of the SiO_2_-embedded polysiloxane coating can be tuned to 1.3523. However, the increase in the number of SiO_2_ nanoparticles negatively affects the mechanical properties and laser damage resistance of the polysiloxane coatings. Nevertheless, the polysiloxane coatings with the prepolymer to SiO_2_-nanoparticles ratio of = 1:80 are considered suitable for practical applications.

### 3.5. Optical Properties of bi-Layer Antireflection Coating System

**Figure 12:**
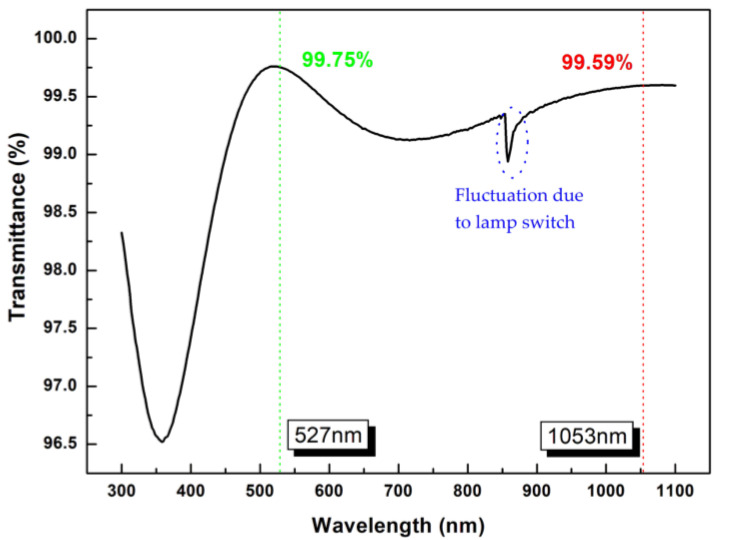
The transmittance of bi-layer antireflection coating system.

A layer of the SiO_2_-embedded polysiloxane coating (prepolymer/SiO_2_ nanoparticle ratio = 1:80) with a thickness of about 130 nm was coated and cured; then, a layer of the SiO_2_ coating (with a thickness of about 70 nm and a refractive index around 1.20) was coated on the cured SiO_2_-embedded polysiloxane coating to prepare a composite bi-layer antireflection coating system. The transmittance of the coating system at 527 nm and 1053 nm reached 98.45% and 98.13%, respectively (Figure 12), indicating that the coating system can realize dual-wavelength antireflection.

## 4. Conclusions

MPS with different degrees of hydrolysis was synthesized using CH_3_COOH as a catalyst and THF as an inhibitor. When the ratio of MPS:H_2_O:THF was 1:4:0 (RT), T_0-1_, T_0-2_ but T_0-3_ was observed in the hydrolysate. When the ratio of MPS:H_2_O:THF was 1:4:1 (IT), the content of T_0-1_ and T_0-3_ was relatively high, and the content of T_0-2_ was lower than that of T_0-1_ and T_0-3_ in the hydrolysate. When the ratio of MPS:H_2_O:THF was 1:4:3 (IT), only T_0-1_ was present in the hydrolysate. Prepolymers were prepared using MPS monomers with different degrees of hydrolysis. It was found that the prepolymer with the reactant ratio of MPS:H_2_O:THF = 1:4:3 (IT) had the highest number of three-dimensional structures. Meanwhile, the prepolymers prepared using hydrolysates from MPS:H_2_O:THF = 1:4:0 (RT) and MPS:H_2_O:THF = 1:4:1 (IT) had more long-chain structures and network structures, respectively. The polysiloxane coatings with different crosslinking structures were prepared using the prepolymers noted above. A three-dimensional crosslinking structure was found to be beneficial for reducing the intrinsic refractive index of the polysiloxane coatings, and a polysiloxane coating with an intrinsic refractive index of 1.485 was fabricated. After adding SiO_2_ nanoparticles, the refractive index of the polysiloxane coating could be precisely adjusted to 1.3732 (when the ratio of prepolymer to SiO_2_-particles was 1:80), and this coating presented the best mechanical properties and laser damage resistance among all the experimental groups. Through the exceptional control of coating thickness, the transmittance of a bi-layer antireflection coating system at 527 nm and 1053 nm reached 99.75% and 99.59%, respectively.

## Figures and Tables

**Table 1:** The concentrations of reactants in different experimental groups.

MPS	H_2_O	THF	CH_3_COOH	Notes
0.1 mol	0.2 mol	/	0.1 mol/L	Effect of catalyst content
0.1 mol	0.2 mol	/	1 mol/L	
0.1 mol	0.2 mol	/	2 mol/L	
0.1 mol	0.1 mol	/	1 mol/L	Effect of water content without THF
0.1 mol	0.2 mol	/	1 mol/L	
0.1 mol	0.3 mol	/	1 mol/L	
0.1 mol	0.4 mol	/	1 mol/L	
0.1 mol	0.4 mol	0.1 mol	1 mol/L	Effect of inhibitor content in water-rich system
0.1 mol	0.4 mol	0.2 mol	1 mol/L	
0.1 mol	0.4 mol	0.3 mol	1 mol/L	
0.1 mol	0.1 mol	0.4 mol	1 mol/L	Effect of water content in THF-rich system
0.1 mol	0.2 mol	0.4 mol	1 mol/L	
0.1 mol	0.3 mol	0.4 mol	1 mol/L	

**Table 2:** The results obtained using different polycondensation methods.

Polycondensation Method	Reactant Ratio of MPS:H_2_O:THF	Polycondensation Product			Refractive Index
		T_2l_	T_2r_	T_3_	
Direct polycondensation	1:4:0 (RT)	Moderate	Strong	Weak	1.4811~1.5049
	1:4:1 (IT)	Moderate	Strong	Weak	1.4917~1.5023
	1:4:3 (IT)	Strong	Strong	Weak	1.4956~1.5080
Polycondensation after the removal of water	1:4:0 (RT)	Moderate	Strong	Moderate	1.4986~1.5006
	1:4:1 (IT)	Moderate	Strong	Moderate	1.5008~1.5030
	1:4:3 (IT)	Moderate	Strong	Weak	1.4802~1.4817
Polycondensation after THF removal	1:4:1 (IT)	Moderate	Strong	Weak	1.5029~1.5049
	1:4:3 (IT)	Moderate	Moderate	Moderate	1.4854~1.4858

**Table 3:** Elastic modulus and hardness of polysiloxane coatings embedded with SiO_2_ nanoparticles.

Ratio of Prepolymer to SiO_2_ Nanoparticles	Elastic Modulus (GPa)	Hardness (MPa)
1:80	5.45	424.91
1:90	3.34	243.65
1:100	2.44	181.82

**Table 4:** LIDT of polysiloxane coatings embedded with SiO_2_ nanoparticles.

Ratio of Prepolymer to SiO_2_ Nanoparticles	9.5 ns Pulsed Laser-Induced Damage Threshold(J/cm^2^)	5 ns Pulsed Laser-Induced Damage Threshold(J/cm^2^)
1:80	74.86	59.79
1:90	70.56	56.36
1:100	54.56	43.58

## Data Availability

The data presented in this study are available on request from the corresponding author. The data are not publicly available due to privacy protection.
